# Factors associated with the duration of symptoms in adult women with suspected cystitis in primary care

**DOI:** 10.1371/journal.pone.0201057

**Published:** 2018-07-25

**Authors:** Mathilde François, Barbara Clais, Thierry Blanchon, Cécile Souty, Thomas Hanslik, Louise Rossignol

**Affiliations:** 1 Université Versailles-Saint-Quentin-en-Yvelines, Versailles, France; 2 Université Paris Saclay, INSERM, Centre de Recherche en Epidémiologie et Santé des Populations, UMR1018, hôpital Paul Brousse, Villejuif, France; 3 Département de médecine générale, Université Paris Descartes, Paris, France; 4 Sorbonne Universités, INSERM, Institut Pierre Louis d’épidémiologie et de Santé Publique (IPLESP UMRS 1136), Paris, France; 5 Hopital Universitaire Ambroise Paré AP-HP, Boulogne-Billancourt, France; Northwestern University, UNITED STATES

## Abstract

**Objective:**

The aim of this study was to identify factors associated to the duration of symptoms of cystitis.

**Patients and methods:**

We conducted a nested survival study using Druti study data. Druti was a cross-sectional survey conducted in adult women visiting a general practitioner in France, for a suspected urinary tract infection between January 2012 and February 2013. For this study, urine cultures were systematically performed for all women. The evolution of symptoms were monitored daily for two weeks. This nested study considered only women with suspected cystitis from Druti; women with pyelonephritis were excluded. To identify independent predictors for duration of symptoms, a Cox proportional hazards regression model was performed.

**Results:**

In Druti, 449 patients had a suspected cystitis. Among them, 440 had a follow up at two weeks. Out of the 440 patients, 424 had a prescription of antibiotic treatment (96.4%). The urine culture was positive for 326 patients (74.1%). The median duration of symptoms after consultation was two days (interquartile 1–3). The absence of urinary frequency (median two days versus three days, p = 0.008), age over 55 years (median two days versus three days, p<0.001) and patient’s bet about the presence of a urinary tract infection (median two days, p = 0.021) were associated to a longer duration of symptoms. Positive culture (p = 0.99) and presence of a multi-drug resistant organism (p = 0.38) did not influence the duration of symptoms.

**Conclusion:**

In a real-life study, factors influencing the duration of symptoms are clinical factors. The delay before re-evaluation in case of persistent symptoms after treatment could be adapted according to the initial clinical examination.

## Introduction

Urinary tract infections (UTIs) are one of the most common bacterial infections with an estimated global incidence of 150,000 million per year [1,2]. In the United States, 10.8% of adult women have had at least one physician-diagnosed suspected UTI over the past year [3]. In France, incidence rate of medical consultation for suspected UTI among women over 18 years old had been estimated at 3,200 per 100,000 women in 2012 [4].

According to French recommendations, the clinical diagnosis for cystitis is based on the presence of voiding symptoms associated or not with the nitrites or leukocytes on urinary dipstick test [5]. The utility of the urinary dipstick test is questioned in the literature, as the likelihood of UTI is over 90–95% when clinical symptoms such as dysuria, urinary frequency without vaginal discharge or irritation are present [6,7]. Likelihood of UTI thus varies according to the combination of clinical symptoms. In the French recommendations, two situations are defined for cystitis: uncomplicated cystitis and cystitis with complication risk [5].

Depending on studies, even without an antibiotic therapy, the evolution of UTI symptoms has been spontaneously favourable in 20–42% of cases in seven to ten days [8–10]. For treated uncomplicated cystitis, the mean and median duration of symptoms ranged between 3.5 days and four days according to previous studies [11–13]. Evolution of symptoms and factors associated with their duration have only been studied for uncomplicated cystitis [8,11,12,14–17]. Factors that increased duration of symptoms were presence of urinary frequency [12], more severe symptoms at baseline [11,12], history of cystitis [12], negative urine cultures [11,12,18], presence of a resistant germ isolate [12,14,16,17,19], no antibiotics prescribed [8,12,15,20,21]. Doctor’s bet about positive diagnosis decreased duration of symptoms [12].

The clinical evolution of symptoms is important to know in order to give better information to patients. The aim of this study was to identify factors influencing the duration of symptoms among women visiting a general practitioner (GP) for a suspected cystitis.

## Materials and methods

### Design and study population

We conducted a nested survival study using Druti study data [4]. Druti was a national cross-sectional prospective survey conducted in France between January 2012 and February 2013 by GPs of a French practice-based surveillance network, the “*Sentinelles”* network [22]. The aim of Druti was to describe the patterns of antibiotic resistance of Enterobacteriaceae involved in community-acquired UTIs.

Patients included were female aged 18 and older visiting their GP for suspected UTI (i.e. complaining of at least one clinical symptom of UTI–dysuria, urinary frequency or urinary urgency) for less than seven days. They had to agree to participate, to avoid any antibiotic during the seven previous days, and to be able to provide a midstream urine sample during the consultation. Population of the Druti study is described elsewhere [4].

For our nested study, a first population was formed, excluding patients with substantial arguments for pyelonephritis (temperature above 38.5°C) [23] or who had not been followed-up to two weeks. From this population, a subgroup, also excluding patients with pelvic or back pain, was formed to rule out any patient who may have pyelonephritis.

### Data collection

For each included patient, GP and patient filled in a questionnaire during the consultation. It included questions regarding the description of the current episode, the bet of the patient and the GP on diagnosis of UTI, the therapeutic management, clinical status (risk factors of complications, previous UTIs…) and patient’s demographic characteristics. For the purposes of the Druti study, a urine sample was collected from all participants, and urine culture performed on all samples at the same laboratory (Ambroise Paré University Hospital, Paris). The GPs and the patients were blinded to the urine culture results.

To recover any missing data, a trained investigator phoned GPs and patients to verify the information, within two weeks of inclusion. A questionnaire was given to the patient at baseline to note the daily evolution of symptoms over the next two weeks. The question was whether patients were still disturbed by their cystitis with a daily report.

English translation of questionnaires used in this study have been published elsewhere [24]. The original version in French is available as Supporting information (S1–S3 Tables).

### Selected variables

Selected variables to identify factors influencing the duration of symptoms were those described in the literature and those deemed relevant by the research team. Selected variables were age [11], clinical symptoms [12], duration of symptoms before consultation of more than three days [12], clinical status (previous UTIs [12], recurrent UTIs, UTI with complication risk), UTI with a suspected resistant germ isolate (i.e.: consultation and antibiotic used by the patient in the previous three months, hospitalisation within 12 months before the study), bet of the patient and the GP on the presence of a UTI [12], results of urine culture (positive urine culture [11,12,18], *E*. *coli*-positive urine culture, multi-resistant isolates [12,14,16,17,19]) and therapeutic management of the episode (antibiotic treatment [8,12,15,20], single-dose antibiotic treatment [21], antibiotic guidelines-compliant in terms of agent used, dose and duration, conferred to the French 2008 recommendations that were the current recommendations in 2012 [25]).

For this analysis, the French definition of cystitis with complication risk was used: cystitis occurring in a woman with urinary tract anomalies, or severe renal failure, or immunocompromising disease / treatment, or pregnancy, or aged 75 years and older or over 65 years old with criterion of fragility [5]. This definition differed from the European and the American definition of complicated cystitis, notably for diabetes mellitus that was no more a risk factor in France [26,27]. Because UTI were more frequent for post-menopausal women, age was included as a two classes variable inspired from literature: under 55 years and over 55 years [28,29]. Recurrent UTI was defined as more than three occurrences in the last year. Urine culture was defined as positive according to the recommendations of the REMIC (microbiology guidelines—bacteriology and mycology, 2010) of the French Society of Microbiology [30]. Multi-drug resistance (MDR) was defined as acquired resistance to at least three antibiotics according to the natural resistance of the isolate [31].

### Statistical analysis

Characteristics of included patients were described as numbers and percentages and quantitative variables as mean (SD) or median (interquartile range, IQR). Survival outcome was defined as the time from consultation to cessation of symptoms within two weeks. Cessation of symptoms was defined as the time (number of days after the initial consultation) where patients were no more inconvenienced by the symptoms. Patients with symptoms at the fifteenth day were censored. Univariate and multivariate analysis was performed with a Cox proportional hazards regression to estimate unadjusted hazards ratios (HRs) and their 95% confidence intervals (95%CIs). Variables selected for multivariate analysis were those achieving a p-value <0.20 in univariate analysis, and those which seemed relevant by the research team and found as predictive in the literature [32].

To avoid introducing strongly correlated variables into multivariate Cox model, we assessed correlations using Cramer’s V for categorical variables and the nonparametric Spearman’s rank correlation for quantitative variables. In case of correlated variables, the most significant variable in univariate analysis or that deemed the most relevant by the research team was introduced in the multivariate analysis. The proportional hazards assumption was tested using Schoenfeld residual plots and tests [33]. We used stratified Cox models to deal with time-dependent variables (i.e., inpatient/outpatient status) [34]. In case of stratified Cox models, interactions were assessed between time-dependent variables and others. Unlike the Druti study, we did not use a sampling design (stratification, stages and sampling weights). Analyses were performed using R software version 2.10.1 [35].

All analysis were performed for both our main population and the subgroup (patients without pelvic or abdominal pain).

### Ethical approval

Before the inclusion, each patient completed a consent form. The data collected were anonymous. Each patient included received a 6-digit anonymity number: the first 4 digits corresponding to the doctor's identifier the last 2 digits corresponding to the patient number (01 for the first patient included). Thus, the third patient included by the doctor number 3760 was anonymized with the number 3760–03.

The study obtained research authorization from the French independent administrative authority protecting privacy and personal data (CNIL), number 911,485 (CCTIRS number 11.474), and from the local human investigation committee of *Ile de France V*.

## Results

### Population

**Fig 1:**
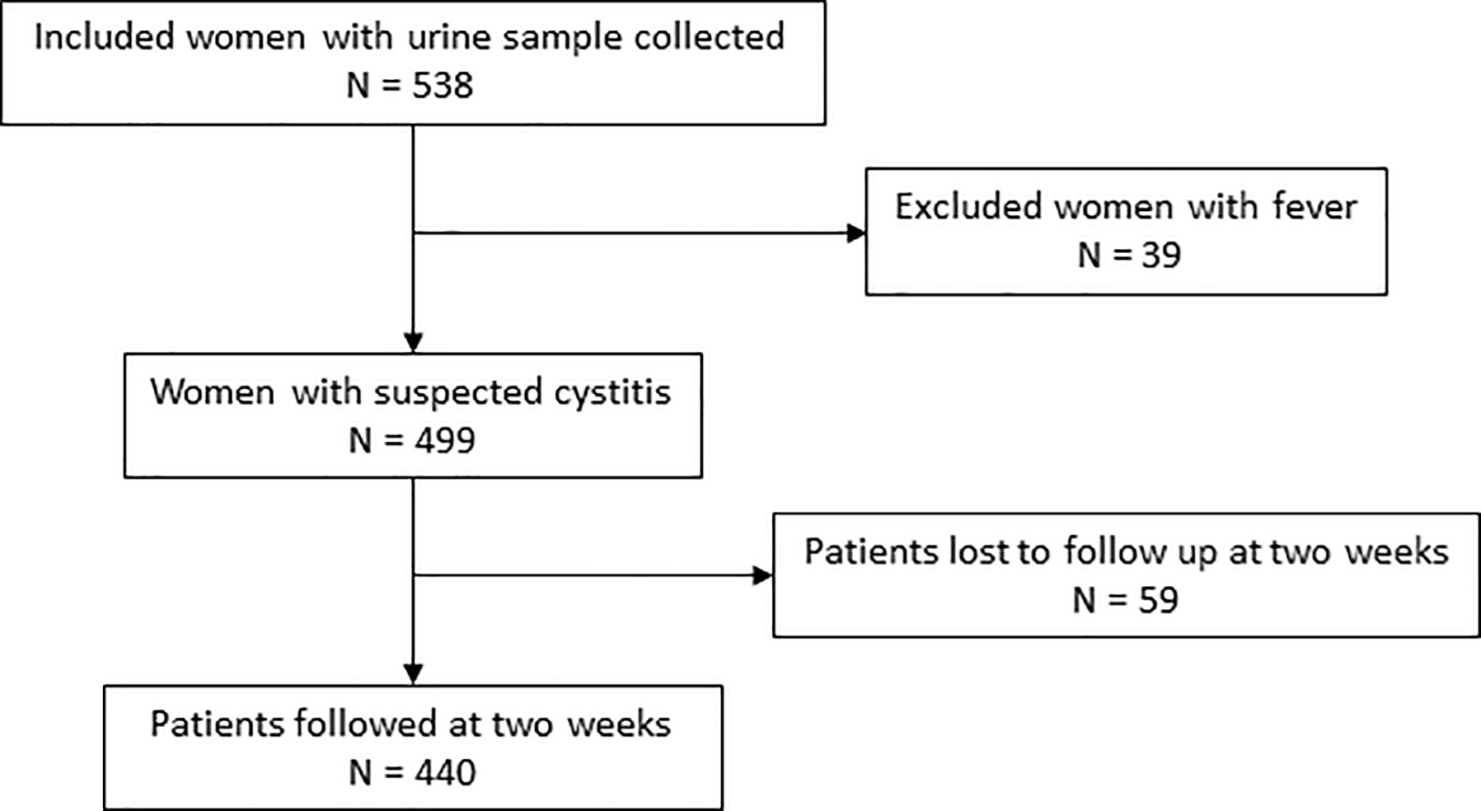
Flow chart.

During the study, 87 GPs participated. Among 538 patients included in the Druti study, only 499 presented a suspected cystitis. Among them, 440 were followed for two weeks (Fig 1). There were 263 patients in the subgroup that were not suffering from pelvic or back pain.

The 440 patients followed and the 59 patients excluded for missing follow up were not different (Table 1).

**Table 1:** Characteristics of patients with suspected cystitis: Patients followed at two weeks (study population) and patients lost to follow up.

	Patients followed N = 440	Patients lost to follow up N = 59	p-value*
**Age (average, min-max in years)**	46 (18–97)	42 (18–86)	0.14
**Dysuria**	415 (94%)	52 (88%)	0.08
**Urinary frequency**	406 (92%)	51 (86%)	0.13
**Urinary urgency**	326 (74%)	37 (63%)	0.13
**Hematuria**	113 (26%)	10 (17%)	0.21
**Pelvic or back pain**	176 (40%)	31 (53%)	0.10
**Vaginal discharge**	26 (6%)	1 (2%)	0.99

* Using Student’s T-test and Chi-Square test

Among the 440 included women, mean age was 46 years (min 18—max 97 years old). Concerning cystitis with complication risk, 59 (13%) women presented a single complication factor, three had two (Table 2).

**Table 2:** Patients with cystitis with complication risk.

Factor of complication risk	Number of patients (% among patients followed)
Pregnancy	8 (2%)
Urinary tract anomalies	18 (4%)
Age > 75 years	32 (7%)
Immunocompromising diseases or treatments	7 (2%)

The average of period between the beginning of UTI symptoms and consultation was 1.9 days (95% CI [1.75;2.05]).

Antibiotic treatment had been received by 424 patients (96.4%). Out of them, 248 had a single dose (56.5%) and 347 received an antibiotic according to the recommendations (Table 3).

**Table 3:** Description of the antibiotic prescribed.

Antibiotic		Dose per tablet	Dosage (number of tablet per day)	Duration of treatment (number of days)	Number of patients treated
Fosfomycin*		**3000**	**1**	**1**	**162 (37%)**
				2	1 (0.2%)
**Nitrofurantoin**		50	2 to 3	2 to 10j	7 (2%)
		100	2	5 to 6	2 (0.5%)
		**100**	**3**	**5**	**2 (0.5%)**
		**100**	**3**	**10**	**1 (0.2%)**
**Fluoroquinolone**					
	**Ciprofloxacin**	**250**	**2**	**3**	**2 (0.2%)**
			2	5 to 10	8 (2%)
		**500**	**1**	**1**	**1 (0.2%)**
			**2**	**5 to 14**	**5 (1%)**
	**Lomefloxacin**	**400**	**1**	**3**	**61 (14%)**
			3	3	1 (0.2%)
	**Norfloxacin**	**400**	1	5	3 (0.7%)
			**2**	**3**	**12 (3%)**
			2	4	2 (0.5%)
			**2**	**5 to 10**	**47 (11%)**
			3	3 to 5	3 (0.7%)
	**Ofloxacin**	**200**	**2**	**5 to 10**	**32 (7%)**
		**400**	**1**	**1**	**3 (0.7%)**
	**Enofloxacin**	**200**	1	5	1 (0.2%)
			2	3	1 (0.2%)
			**2**	**5 to 10**	**4** (0.9%)
			3	8	1 (0.2%)
Quinolone					
	Pefloxacin	800	1	1	6 (1%)
	Pipemidic acid	400	1 to 2	5 to 10	4 (0.9%)
Third generation cephalosporin					
	**Cefixime**	200	2	3 to 4	4 (0.9%)
		**200**	**2**	**5 to 10**	**15 (3%)**
	Cefpodoxime	100	2	5 to 10	7 (2%)
	Ceftriaxone	1000	1	1	1 (0.2%)
Penicillin					
	Amoxicillin	1000	2 to 3	5 to 15	12 (3%)
	Amoxicillin/clavulanic acid	1000	2 to 3	5 to 10	6 (1%)
trimethoprim sulfamethoxazole		400	2 to 3	5 to 6	3 (0.7%)
		800	2	3 to 8	9 (2%)
		1200	1	1	1 (0.2%)

* Bolt text represent antibiotic guideline-compliant in term of agent used, dose, dosage and duration Urine culture was positive for 326 samples: 275 with *E*. *coli* and 61 with MDR isolates.

### Duration of symptoms and factors associated

The median duration of symptoms since the inclusion consultation was two days (IQR 1–3), 106 still had symptoms at the fourth day (24%) and 34 at two weeks (6.8%).

After univariate analysis, only clinical variables presented a *p-value* under 0.20 (Table 4).

**Table 4:** Factors associated to the duration of symptoms for women with suspected cystitis: Univariate analysis.

Selected variables		Patients (%)	Median duration of symptoms (days)^a^	Hazards Ratio [95%CI]	*p value*^b^
**Dysuria**	Yes	415 (94.3)	2	0.99 [0,66–1,52]	0.99
	No	25 (5.7)	2		
**Urinary frequency**	Yes	406 (92.3)	2	1.60 [1,9–2,35]	**0.02**
	No	34 (7.7)	3		
**Urinary urgency**	Yes	326 (74)	2	1.06 [0.85–1.33]	0.59
	No	114 (26)	2		
**Hematuria**	Yes	113 (25.7)	2	1.06 [0.85–1.32]	0.61
	No	326 (74.3)	2		
**Pelvic or back pain**	Yes	176 (40)	2	0.83 [0.68–1.02]	0.07
	No	263 (60)	2		
**Vaginal discharge**	Yes	413 (64)	1.5	1.30 [0.86–1.95]	0.22
	No	26 (6)	2		
**Cystitis with complication risk**^**c**^	Yes	62 (14.1)	3	0.71 [0.53–0.95]	**0.02**
	No	377 (85.9)	2		
**Age ≥ 55 years old**	Yes	145 (33)	3	0.71 [0.57–0.87]	**<0.01**
	No	295 (67)	2		
**Previous UTI**^**d**^	Yes	375 (85)	3	0.85 [0.65–1.12]	0.25
	No	65 (15)	2		
**Recurrent UTI**^**e**^	Yes	34 (7.7)	3	0.87 [0.61–1.24]	0.45
	No	405 (92.3)	2		
**Antibiotic use by the patient in the previous three months**	Yes	349 (79.3)	2	1.07 [0.84–1.35]	0.61
	No	91 (20.7)	2		
**Hospitalization in the previous 12 months**	Yes	48 (10.9)	2	1.04 [0.76–1.42]	0.83
	No	392 (89.1)	2		
**Consultation in the previous three months**	Yes	295 (67)	2	0.83 [0.67–1.01]	0.07
	No	145 (33)	2		
**Period before consultation longer than three days**	Yes	70 (15.9)	3	0.81 [0.62–1.05]	0.11
	No	370 (84.1)	2		
**GP’s bet on a positive urine culture**	Yes	401 (91.6)	2	0.77 [0.55–1.10]	0.15
	No	37 (8.4)	2		
**Patient’s bet on a positive urine culture**	Yes	423 (96.8)	2	0.60 [0.35–1.03]	**0.06**
	No	14 (3.2)	2		
**Positive urine culture**	Yes	326 (74.1)	2	1.00 [0.80–1.25]	0.99
	No	114 (25.9)	2		
**UTI due to *E*. *coli***	Yes	275 (62.5)	2	0.92 [0.75–1.12]	0.40
	No	165 (37.5)	2		
**MDR**^**f**^ **isolates**	Yes	61 (13.9)	3	0.88 [0.66–1.17]	0.38
	No	379 (86.1)	2		
**Antibiotic**	Yes	424 (96.4)	3	0,71 [0.16–1.26]	0.37
	No	16 (3.6)	2		
**Single dose antibiotic treatment**	Yes	248 (56.5)	2	1.30 [1.06–1.58]	**0.01**
	No	176 (41.5)	3		
**Antibiotic guideline-compliant**	Yes	347 (81%)	2	1.07 [0.82–1.40]	0.61
	No	83 (19%)	2		

^a^ since the inclusion consultation

^b^Univariate cox models

^c^ Cystitis with complication risk was defined as cystitis occuring in a woman with urinary tract anomalies, or severe renal failure, or immunocompromising disease/treatment, or pregnancy, or aged 75 years and older or over 65 years old with criteron of fragility;

^d^ UTI: Urinary tract infection

^e^ Patients with ≥ four UTIs/year

^f^ MDR: multidrug resistance defined as acquired resistance to at least three of antimicobial antibiotics according to the natural resistance of the isolate.

Positive urine cultures and multi-drug resistance of *E*. *Coli* were forced into the multivariate model.

As age was one factor used for the definition of cystitis with complication risk, the correlation risk between these both variables was very high. Therefore keeping both of them in the multivariate analysis was not recommended and only age was kept. Significant correlations were also found between GP’s bet and patient’s bet (p = 0.004), consultation in the previous three months and age (p<0.001), consultation in the previous three months and cystitis with complication risk (p = 0,008). Therefore, we kept age and patient’s bet for the multivariate analysis.

The variable single dose antibiotic treatment did not meet the proportional hazards assumption (p < 0.001). So the model was stratified on this variable. No interaction between this variable and other variables in the model were found.

The final model is presented in Table 5. Presence of urinary frequency significantly reduced the duration of symptoms (median duration: two days vs three days, p = 0.008). Age ≥ 55 years old (median duration: three days vs two days, p < 0.001) and patient’s bet on a positive urine culture (median duration: two days for both, p = 0.021) increased the duration of symptoms (Table 5).

**Table 5:** Factors associated to the duration of symptoms for women with suspected cystitis: Multivariate analysis.

	Hazards Ratio [95%CI]	*p value*
**Age ≥ 55 years old**	0.68 [0.55–0.85]	**< 0.001**
**Presence of urinary frequency**	1.70 [1.15–2.52]	**0.008**
**Patient’s bet on a positive urine culture**	0.52 [0.30–0.90]	**0.02**

For the subgroup of patients without pelvic or back pain, no predictor of healing time has been identified: results were not significant (data not shown).

## Discussion

This is the first study which investigated factors predicting duration of symptoms for women with suspected cystitis including cystitis with complication risk. In these patients attending in primary care, the median duration of symptoms was two days since the inclusion consultation. Presence of a urinary frequency significantly reduced the duration of symptoms, an age 55 years and older and patient’s bet on a positive urine culture increased this duration.

The duration of symptoms measured here was shorter than expected. In the literature, the mean and median duration of symptoms of treated uncomplicated cystitis were 3.5 days and four days respectively [11–13]. As in our study, the start date was the consultation date, corresponding to the beginning of antibiotic therapy. In two of these three studies, fewer patients received antibiotics than in our study [12,13]. Prescription of antibiotic reduces the duration of symptoms, while there was no significant differences between the different antibiotic management strategies [11–13]. Similary, contrary to our study, the presence of urinary frequency increased duration of symptoms in the literature [12]. Urinary frequency is probably the most troublesome symptom, impacting directly on the organization of everyday life. During the monitoring carried out, the question was "Are you still inconvenienced?" without specifically targeting each symptom. Thus, some patients with this disabling symptom reduction could answer "no" without being completely healed.

Regarding age, a previous Belgian study found no difference in the duration of symptoms between patients over 55 years and patients under 55 years (p = 0.984) [11] but this study excluded cystitis with complication risk. Among our patients aged 55 years and older, 30% have a UTI with complication risk while only 6% of patients under 55 years had one. The presence of cystitis with complication risk, which significantly increased the duration of symptoms in our univariate analysis, may explain this difference. This variable could not have been included in multivariate analyse because of its correlation with age. Further studies are needed to conclude on the respective share of age and the presence of complication risk.

The duration of symptoms was significantly increased when patient thought that they had a positive urine culture. Patients, who self-diagnose and try self-medication, consulted only when symptoms persist, worsen, cause confusion in everyday life, or when there was fear of complications [36,37]. Perhaps, these patients have initially more severe symptoms and the duration of symptoms had been shown to be longer in this case [11,12].

In our study, positive urine culture does not influence the duration of symptoms. This result is similar to a Norwegian study [18] but contradicts other studies [11,12] for which the duration was shorter with a positive culture. The presence of MDR was not associated with the duration of symptoms, unlike what is found in the literature [14,16,17,19]. However, even if the germ was resistant to the prescribed antibiotic, the absence of a relationship between clinical and bacteriological results was found in other studies, where the eradication rate after treatment was not related to the clinical cure rate [17,19].

Because antibiotic treatment did not meet the proportional hazards assumption, we cannot conclude about its effect on duration of symptoms with the multivariate analysis.

The population of the study were women visiting their GP for suspected UTI. Women with suspected UTI who did not consult a GP (e.g., visit to another specialist, self-medication or spontaneous healing) were not taken into account. Indeed, a US study estimated that only 50% of UTIs had a medical visit [38]. The number of UTI (499) in our study could seem low given the number of GPs (87). However, it corresponded to patients that agreed to be included, that were followed two weeks after inclusion and that did not have fever. Another study from Druti has shown that the incidence of UTI in this population was consistent with the literature [39].

Our study did not take into account the severity of symptoms and the general symptoms (unwell, feverish…), which are usually present in up to two thirds of patients [11]. The presence of general symptoms would not have changed the median duration of symptoms because these signs were the first to disappear (median two days) [11]. In the literature, initial severe symptoms were correlated with a longer duration of symptoms [11,12]. In practice, there was no standardized assessment of the severity of symptoms in 2012 [40]. Each study about efficacy of antibiotic on UTI used its own questionnaire, not always described [41,42]. The initial assessment and re-evaluation of patients with suspected cystitis is not included in the French guidelines [5]. In this study, the question for the follow-up was "Are you still inconvenienced?", estimating an overall healing time. The duration of each voiding symptom could not be determined. However, the most important information is the overall healing sensation of patients rather than the evolution of each symptom. Contrary to the European guidelines, the French guidelines did not considered the previous treatment failure as a complicated UTI [5,26]. This criteria, which appears during the follow-up, was not considered in our study, as in the other [11–13]. At last, the complication factor "women aged between 65 and 75 years with criterion of fragility" could not have been taken into account for lack of data. Due to its correlation with age, the risk of complication was not used in the multivariate model. The lack of this data did not had impact on our results. Data collection had not taken into account the difference between back pain and pelvic pain. This could lead to the inclusion of patients suffering from pyelonephritis in our main population. This is why we performed a sub-group analysis without patients suffering from pelvic or back pain. Results of this analysis were not significant, probably due to the lack of power. However, incidence of pyelonephritis is considerably lower than incidence of cystitis: for women with recurrent infection, the ratio of episodes of acute pyelonephritis to episodes of acute cystitis has been reported to be 1:18 and 1:29 [43–45]. Although back pain is suggestive of pyelonephritis [46], near 50% of cystitis had lower back pain in a Belgian study [11]. Furthermore, patients with fever were excluded. Although lack of fever is common in the elderly, only 32 patients were 75 or older in our study [47]. Thus, despite maintaining the variable “pelvic or back pain” in our main population, there should be only a few patients with pyelonephritis included. This must be taken into account when analysing our results. Another symptoms like flank or abdominal pain, nausea or vomiting might occur during a pyelonephritis but the presence of these symptoms were not available in the database, preventing the distinction of pyelonephritis from cystitis in this way. Therefore in our study, the definition of pyelonephritis is limited to the presence of a temperature above 38.5°C. The presence of vaginal discharge or irritation decreases the likelihood of UTI in some studies [6,7]. However, a more recent meta-analysis concluded that vaginal discharge was a weak predictor of absence of infection [48]. This is why we preferred not to exclude these patients.

This study has several strengths. To our knowledge, this study is one of the first to systematically collect urine samples with centralized analysis from all women presenting with symptoms of a UTI in general practice. It is also the first to include cystitis with complication risk. GP did not use a standardized treatment, but continued to apply their usual patient-centered practice. This permitted to have data from the actual daily practice of GPs. Another strength was in the standardized collection of data, which limited information bias. Lastly, our study population is similar to the literature for the average age, the frequency of dysuria and urinary frequency [8,49,50]. The urinary urgency and pelvic pain were less frequent, the hematuria more frequent [8,11,14,51]. The urine culture was positive in 74.1% of patients, with 84% to *E*. *coli*, which is comparable to studies including women with suspected uncomplicated cystitis (respectively 64.2% to 74.6% and 75 to 95%) [12,51,52].

## Conclusion

The predictive factors identified were clinical. There was no relationship between the evolution of symptoms and bacteriological results. According to the European and American guidelines concerning uncomplicated cystitis, a urine culture should be performed if symptoms do not resolve by the end of the treatment (ranging from one to five days) [26,27]. The French guidelines require informing patients that symptoms may persist for two to three days after treatment and that they may benefit from a urinary culture if their symptoms persist more than three days, whatever the initial clinical presentation is. In our study, one quarter of the patients remain symptomatic on the fourth day which means that one patient out of four should have a urine test according to the French guidelines. As predictive factors identified were clinical, this result could be taken into account for guidelines to perform a urine sample if symptoms persist after considering the initial clinical examination. This approach may be useful to the clinicians to adjust their speech to patient about the evolution of symptoms and the time before collecting a urine sample: be more patient with patients aged 55 and over and less patient for patients with urinary frequency.

## Supporting information

S1 Table Registry of consultations for urinary tract infection (in French). (DOC)

S2 Table Inclusion questionnaire (in French). (DOC)

S3 Table Follow-up questionnaire (in French). (DOC)

## References

[1] Foxman B. Epidemiology of urinary tract infections: Incidence, morbidity, and economic costs. Dis Mon. 2003;49: 53–70. 10.1067/mda.2003.7 12601337

[2] Harding GK, Ronald AR. The management of urinary infections: what have we learned in the past decade? Int J Antimicrob Agents. 1994;4: 83–88. 18611593 10.1016/0924-8579(94)90038-8

[3] Foxman B, Barlow R, D’Arcy H, Gillespie B, D. Sobel J. Urinary tract infection: self reported incidence and associated costs. Ann Epidemio. 2000;10: 509–515. 11118930 10.1016/s1047-2797(00)00072-7

[4] Rossignol L, Maugat S, Blake A, Vaux S, Heym B, Le Strat Y, et al Risk factors for resistance in urinary tract infections in women in general practice: A cross-sectional survey. J Infect. 2015; 10.1016/j.jinf.2015.05.012 26054878

[5] Société de Pathologie Infectieuse de Langue Française. Diagnostic et antibiothérapie des infections urinaires bactériennes communautaires de l’adulte. 2014.

[6] Bent S, Nallamothu BK, Simel DL, Fihn SD, Saint S. Does this woman have an acute uncomplicated urinary tract infection? JAMA J Am Med Assoc. 2002;287: 2701–2710. 10.1001/jama.287.20.2701 12020306

[7] Guay DRP. Contemporary management of uncomplicated urinary tract infections. Drugs. 2008;68: 1169–1205. 18547131 10.2165/00003495-200868090-00002

[8] Ferry SA, Holm SE, Stenlund H, Lundholm R, Monsen TJ. Clinical and bacteriological outcome of different doses and duration of pivmecillinam compared with placebo therapy of uncomplicated lower urinary tract infection in women: the LUTIW project. Scand J Prim Health Care. 2007;25: 49–57. 10.1080/02813430601183074 17354160 PMC3389454

[9] Ferry SA, Holm SE, Stenlund H, Lundholm R, Monsen TJ. The natural course of uncomplicated lower urinary tract infection in women illustrated by a randomized placebo controlled study. Scand J Infect Dis. 2004;36: 296–301. 15198188 10.1080/00365540410019642

[10] Christiaens TCM, De Meyere M, Verschraegen G, Peersman W, Heytens S, De Maeseneer JM. Randomised controlled trial of nitrofurantoin versus placebo in the treatment of uncomplicated urinary tract infection in adult women. Br J Gen Pract J R Coll Gen Pract. 2002;52: 729–734. PMC1314413 12236276

[11] Heytens S, De Sutter A, De Backer D, Verschraegen G, Christiaens T. Cystitis: symptomatology in women with suspected uncomplicated urinary tract infection. J Womens Health 2002. 2011;20: 1117–1121. 10.1089/jwh.2010.2302 21671766

[12] Little P, Merriman R, Turner S, Rumsby K, Warner G, Lowes JA, et al Presentation, pattern, and natural course of severe symptoms, and role of antibiotics and antibiotic resistance among patients presenting with suspected uncomplicated urinary tract infection in primary care: observational study. BMJ. 2010;340: b5633 10.1136/bmj.b5633 20139213 PMC2817050

[13] Little P, Moore MV, Turner S, Rumsby K, Warner G, Lowes JA, et al Effectiveness of five different approaches in management of urinary tract infection: randomised controlled trial. BMJ. 2010;340: c199 10.1136/bmj.c199 20139214 PMC2817051

[14] McNulty C a. M, Richards J, Livermore DM, Little P, Charlett A, Freeman E, et al Clinical relevance of laboratory-reported antibiotic resistance in acute uncomplicated urinary tract infection in primary care. J Antimicrob Chemother. 2006;58: 1000–1008. 10.1093/jac/dkl368 16998209

[15] Falagas ME, Kotsantis IK, Vouloumanou EK, Rafailidis PI. Antibiotics versus placebo in the treatment of women with uncomplicated cystitis: a meta-analysis of randomized controlled trials. J Infect. 2009;58: 91–102. 10.1016/j.jinf.2008.12.009 19195714

[16] Butler CC, Hillier S, Roberts Z, Dunstan F, Howard A, Palmer S. Antibiotic-resistant infections in primary care are symptomatic for longer and increase workload: outcomes for patients with E. coli UTIs. Br J Gen Pract J R Coll Gen Pract. 2006;56: 686–692. PMC1876635 16954001

[17] Abrahamian FM, Krishnadasan A, Mower WR, Moran GJ, Coker JR, Talan DA. The association of antimicrobial resistance with cure and quality of life among women with acute uncomplicated cystitis. Infection. 2011;39: 507–514. 10.1007/s15010-011-0163-z 21789523

[18] Baerheim A, Digranes A, Hunskaar S. Equal symptomatic outcome after antibacterial treatment of acute lower urinary tract infection and the acute urethral syndrome in adult women. Scand J Prim Health Care. 1999;17: 170–173. 10555247 10.1080/028134399750002593

[19] Raz R, Chazan B, Kennes Y, Colodner R, Rottensterich E, Dan M, et al Empiric use of trimethoprim-sulfamethoxazole (TMP-SMX) in the treatment of women with uncomplicated urinary tract infections, in a geographical area with a high prevalence of TMP-SMX-resistant uropathogens. Clin Infect Dis Off Publ Infect Dis Soc Am. 2002;34: 1165–1169. 10.1086/339812 11941541

[20] Knottnerus BJ, Geerlings SE, Moll van Charante EP, ter Riet G. Women with symptoms of uncomplicated urinary tract infection are often willing to delay antibiotic treatment: a prospective cohort study. BMC Fam Pract. 2013;14: 71 10.1186/1471-2296-14-71 23721260 PMC3671219

[21] Falagas ME, Vouloumanou EK, Togias AG, Karadima M, Kapaskelis AM, Rafailidis PI, et al Fosfomycin versus other antibiotics for the treatment of cystitis: a meta-analysis of randomized controlled trials. J Antimicrob Chemother. 2010;65: 1862–1877. 10.1093/jac/dkq237 20587612

[22] Flahault A, Blanchon T, Dorléans Y, Toubiana L, Vibert JF, Valleron AJ. Virtual surveillance of communicable diseases: a 20-year experience in France. Stat Methods Med Res. 2006;15: 413–421. 10.1177/0962280206071639 17089946

[23] Collège universitaire des enseignants de néphrologie (France), Moulin B, Peraldi M-N. Néphrologie. Paris: Ellipses; 2012.

[24] François M, Hanslik T, Dervaux B, Le Strat Y, Souty C, Vaux S, et al The economic burden of urinary tract infections in women visiting general practices in France: a cross-sectional survey. BMC Health Serv Res. 2016;16: 365 10.1186/s12913-016-1620-2 27507292 PMC4977873

[25] Afssaps. Diagnostic et antibiothérapie des infections urinaires bactériennes communautaires chez l’adulte. 2008. Available from: http://www.esculape.com/uronephro/infections-urinaire-adulte-afssaps2008.pdf 19209393

[26] Grabe M, Bartoletti R, Bjerklund-Johanson T., Cai T, Çek M, Köves B, et al Guidelines on urological infections. European Association of Urology; 2015.

[27] Colgan R, Williams M. Diagnosis and treatment of acute uncomplicated cystitis. Am Fam Physician. 2011;84: 771–776. 22010614

[28] Greendale GA, Lee NP, Arriola ER. The menopause. Lancet Lond Engl. 1999;353: 571–580. 10.1016/S0140-6736(98)05352-5 10028999

[29] Savonitto S, Colombo D, Franco N, Misuraca L, Lenatti L, Romano IJ, et al Age at Menopause and Extent of Coronary Artery Disease Among Postmenopausal Women with Acute Coronary Syndromes. Am J Med. 2016;129: 1205–1212. 10.1016/j.amjmed.2016.05.031 27321972

[30] Société Française de Microbiologie. Rémic—Référentiel en microbiologie médicale 2010 - 4ème édition Société française de microbiologie; 2010.

[31] Magiorakos A-P, Srinivasan A, Carey RB, Carmeli Y, Falagas ME, Giske CG, et al Multidrug-resistant, extensively drug-resistant and pandrug-resistant bacteria: an international expert proposal for interim standard definitions for acquired resistance. Clin Microbiol Infect Off Publ Eur Soc Clin Microbiol Infect Dis. 2012;18: 268–281. 10.1111/j.1469-0691.2011.03570.x 21793988

[32] Boëlle P-Y. Multivariate methods (3): Cox’s regression. Sang Thromb Vaiss. 1999;11: 45–50.

[33] Samson E. Identification des marqueurs pronostiques chez les patients atteints d’un cancer de la tête et du cou. Département de mathématiques et statistique—Université Laval, Québec; 2008.

[34] Timsit J-F, Alberti C, Chevret S. Le modèle de Cox. Rev Mal Respir. 2005: 1058–64. 10.1019/200530109 16227945

[35] Team RC. R: A Language and Environment for Statistical Computing (R Foundation for Statistical Computing, Vienna, 2012). URL http://www.R-Proj.Org. 2015;

[36] Leydon GM, Turner S, Smith H, Little P, UTIS team. The journey from self-care to GP care: a qualitative interview study of women presenting with symptoms of urinary tract infection. Br J Gen Pract J R Coll Gen Pract. 2009;59: e219–225. 10.3399/bjgp09X453459 19566988 PMC2702035

[37] Leydon GM, Turner S, Smith H, Little P, UTIS team. Women’s views about management and cause of urinary tract infection: qualitative interview study. BMJ. 2010;340: c279 10.1136/bmj.c279 20139217 PMC2817049

[38] Keating KN, Perfetto EM, Subedi P. Economic burden of uncomplicated urinary tract infections: direct, indirect and intangible costs. Expert Rev Pharmacoecon Outcomes Res. 2005;5: 457–466. 10.1586/14737167.5.4.457 19807263

[39] Rossignol L, Vaux S, Maugat S, Blake A, Barlier R, Heym B, et al Incidence of urinary tract infections and antibiotic resistance in the outpatient setting: a cross-sectional study. Infection. 2016; 10.1007/s15010-016-0910-2 27234045

[40] Holm A, Cordoba G, Siersma V, Brodersen J. Development and validation of a condition-specific diary to measure severity, bothersomeness and impact on daily activities for patients with acute urinary tract infection in primary care. Health Qual Life Outcomes. 2017;15: 57 10.1186/s12955-017-0629-5 28340586 PMC5366156

[41] Bollestad M, Grude N, Lindbaek M. A randomized controlled trial of a diagnostic algorithm for symptoms of uncomplicated cystitis at an out-of-hours service. Scand J Prim Health Care. 2015;33: 57–64. 10.3109/02813432.2015.1041827 25961367 PMC4834504

[42] Hooton TM, Roberts PL, Stapleton AE. Cefpodoxime vs Ciprofloxacin for Short-Course Treatment of Acute Uncomplicated Cystitis. JAMA J Am Med Assoc. 2012;307: 583–589. 10.1001/jama.2012.80 22318279 PMC3736973

[43] Nicolle LE. Empirical treatment of acute cystitis in women. Int J Antimicrob Agents. 2003;22: 1–6. 12842322 10.1016/s0924-8579(03)00101-8

[44] Stamm WE, McKevitt M, Roberts PL, White NJ. Natural History of Recurrent Urinary Tract Infections in Women. Rev Infect Dis. 1990;13: 77–84. 10.1093/clinids/13.1.77 2017637

[45] Ikäheimo R, Siitonen A, Heiskanen T, Kärkkäinen U, Kuosmanen P, Lipponen P, et al Recurrence of urinary tract infection in a primary care setting: analysis of a 1-year follow-up of 179 women. Clin Infect Dis Off Publ Infect Dis Soc Am. 1996;22: 91–99. 10.1093/clinids/22.1.91 8824972

[46] Kinouani S, de Lary de Latour H, Joseph J-P, Letrilliart L. Diagnostic strategies for urinary tract infections in French general practice. Med Mal Infect. 2017;47: 401–408. 10.1016/j.medmal.2017.05.003 28606664

[47] Lane DR, Takhar SS. Diagnosis and management of urinary tract infection and pyelonephritis. Emerg Med Clin North Am. 2011;29: 539–552. 10.1016/j.emc.2011.04.001 21782073

[48] Medina-Bombardó D, Jover-Palmer A. Does clinical examination aid in the diagnosis of urinary tract infections in women? A systematic review and meta-analysis. BMC Fam Pract. 2011;12: 111 10.1186/1471-2296-12-111 21985418 PMC3207883

[49] Medina-Bombardó D, Seguí-Díaz M, Roca-Fusalba C, Llobera J, dysuria team. What is the predictive value of urinary symptoms for diagnosing urinary tract infection in women? Fam Pract. 2003;20: 103–107. 12651780 10.1093/fampra/20.2.103

[50] Baerheim A, Digranes A, Jureen R, Malterud K. Generalized symptoms in adult women with acute uncomplicated lower urinary tract infection: an observational study. Medscape Gen Med. 2003;5: 1. 14600638

[51] Neuzillet Y, Naber KG, Schito G, Gualco L, Botto H. French results of the ARESC study: clinical aspects and epidemiology of antimicrobial resistance in female patients with cystitis. Implications for empiric therapy. Médecine Mal Infect. 2012;42: 66–75. 10.1016/j.medmal.2011.07.005 22264668

[52] Naber KG, Schito G, Botto H, Palou J, Mazzei T. Surveillance study in Europe and Brazil on clinical aspects and Antimicrobial Resistance Epidemiology in Females with Cystitis (ARESC): implications for empiric therapy. Eur Urol. 2008;54: 1164–1175. 10.1016/j.eururo.2008.05.010 18511178

