# Supplementary material for: Factors associated with the duration of symptoms in adult women with suspected cystitis in primary care
Source: PLoS One. 2018 Jul 25;13(7):e0201057. doi: 10.1371/journal.pone.0201057 (PMC6059455; doi:10.1371/journal.pone.0201057)
Supplement: S1 Table — (DOC) [file pone.0201057.s001.doc]

# Etude DRUTI - Registre des consultations pour infection urinaire

L’étude Druti cible les femmes consultant en médecine générale pour suspicion d’infection urinaire. Les critères d’éligibilité et d’inclusion à cette étude sont rappelés ci-dessous et sur le schéma d’inclusion. Ce registre est nécessaire au redressement des estimations d’incidence. Merci de bien vouloir compléter une ligne pour chaque patiente vous consultant pour une infection urinaire répondant aux critères d’éligibilité ci-dessous, même si elle ne participe finalement pas à l’étude pour quelque motif que ce soit.

**Critères d’éligibilité : Femmes, âgées de 18 ans ou plus, non-institutionnalisées, présentant au moins un des symptômes suivants depuis une durée ≤ 7 jours (brûlures ou douleur mictionnelles, pollakiurie, mictions impérieuses) et en l’absence de prurit vulvaire et de pertes vaginales ; et, ayant une bonne compréhension de la langue française et en l’absence de troubles cognitifs, afin de permettre la participation de la patiente à l’étude et le remplissage du questionnaire ; et n’ayant pas été incluse dans l’étude au cours des 8 dernières semaines.**

**Critères d’inclusion : Patientes présentant l’ensemble des critères d’éligibilité, consultant un lundi, mardi, mercredi ou jeudi (jeudi exclu pour la Corse), ayant accepté de participer à l’étude, et ayant réalisé un prélèvement d’urine lors de la consultation.**

**Critères de non inclusion : les patientes ayant reçu une antibiothérapie au cours des 7 derniers jours**

✄ A découper selon les pointillés avant envoi au R Sentinelles

| **Num** | **Age** | **Date de consultation** | **Signes cliniques** | | | **Inclusion** | **Motif de non inclusion**  **(merci de cocher une case)** | **Inclusion** | **Date d’expédition** | **Nom et prénom**  **+ N° d’anonymat** |
| --- | --- | --- | --- | --- | --- | --- | --- | --- | --- | --- |
|  |  |  |  | **Oui** | **Non** |  |  |  |  |  |
| 1 | _ _ | _ _ / _ _ / _ _ | Brûlures / Douleurs à la miction | ❒ | ❒ | ❒ Non | ❒ **antibiothérapie au cours des 7 derniers jours**  ❒ prescription antérieure ❒ délivrance récente  ❒ refus de la patiente  ❒ prélèvement urinaire non réalisé  ❒ jours de consultation ne permettant pas un envoi en 48h (V et S, et en plus J si Corse)  ❒ manque de temps / trop de patients à recevoir  ❒ autre : ………………………………………… | **❒ Oui** | _ _/_ _/_ _ _ _ | ______________  ______________  /_ _ _ _/- /_ _/ |
|  |  |  | Pollakiurie | ❒ | ❒ |  |  |  |  |  |
|  |  |  | Impériosité mictionnelle | ❒ | ❒ |  |  |  |  |  |
|  |  |  | Hématurie | ❒ | ❒ |  |  |  |  |  |
|  |  |  | Douleurs pelviennes / lombaires | ❒ | ❒ |  |  |  |  |  |
|  |  |  | Fièvre (>38,5°Celsius) | ❒ | ❒ |  |  |  |  |  |
|  |  |  | Prurit / Pertes vaginales | ❒ | ❒ |  |  |  |  |  |
| 2 | _ _ | _ _ / _ _ / _ _ | Brûlures / Douleurs à la miction | ❒ | ❒ | ❒ Non | ❒ **antibiothérapie au cours des 7 derniers jours**  ❒ prescription antérieure ❒ délivrance récente  ❒ refus de la patiente  ❒ prélèvement urinaire non réalisé  ❒ jours de consultation ne permettant pas un envoi en 48h (V et S, et en plus J si Corse)  ❒ manque de temps / trop de patients à recevoir  ❒ autre : ………………………………………… | **❒ Oui** | _ _/_ _/_ _ _ _ | ______________  ______________  /_ _ _ _/- /_ _/ |
|  |  |  | Pollakiurie | ❒ | ❒ |  |  |  |  |  |
|  |  |  | Impériosité mictionnelle | ❒ | ❒ |  |  |  |  |  |
|  |  |  | Hématurie | ❒ | ❒ |  |  |  |  |  |
|  |  |  | Douleurs pelviennes / lombaires | ❒ | ❒ |  |  |  |  |  |
|  |  |  | Fièvre (>38,5°Celsius) | ❒ | ❒ |  |  |  |  |  |
|  |  |  | Prurit / Pertes vaginales | ❒ | ❒ |  |  |  |  |  |
| 3 | _ _ | _ _ / _ _ / _ _ | Brûlures / Douleurs à la miction | ❒ | ❒ | ❒ Non | ❒ **antibiothérapie au cours des 7 derniers jours**  ❒ prescription antérieure ❒ délivrance récente  ❒ refus de la patiente  ❒ prélèvement urinaire non réalisé  ❒ jours de consultation ne permettant pas un envoi en 48h (V et S, et en plus J si Corse)  ❒ manque de temps / trop de patients à recevoir  ❒ autre : ………………………………………… | **❒ Oui** | _ _/_ _/_ _ _ _ | ______________  ______________  /_ _ _ _/- /_ _/ |
|  |  |  | Pollakiurie | ❒ | ❒ |  |  |  |  |  |
|  |  |  | Impériosité mictionnelle | ❒ | ❒ |  |  |  |  |  |
|  |  |  | Hématurie | ❒ | ❒ |  |  |  |  |  |
|  |  |  | Douleurs pelviennes / lombaires | ❒ | ❒ |  |  |  |  |  |
|  |  |  | Fièvre (>38,5°Celsius) | ❒ | ❒ |  |  |  |  |  |
|  |  |  | Prurit / Pertes vaginales | ❒ | ❒ |  |  |  |  |  |
