# Supplementary material for: Factors associated with the duration of symptoms in adult women with suspected cystitis in primary care
Source: PLoS One. 2018 Jul 25;13(7):e0201057. doi: 10.1371/journal.pone.0201057 (PMC6059455; doi:10.1371/journal.pone.0201057)
Supplement: S2 Table — (DOC) [file pone.0201057.s002.doc]

**Caractéristiques socio-économiques**

*Cette partie peut être remplie directement par la patiente, pendant que le médecin conditionne le prélèvement urinaire.*

Nous tenons à vous remercier pour votre participation à notre étude. Pour compléter les réponses que vous avez fournies à votre médecin, merci de répondre aux questions suivantes afin d’évaluer l’impact économique des infections urinaires et de décrire les caractéristiques sociales des patientes concernées. Vos réponses resteront strictement confidentielles et anonymes.

| 38. Vivez-vous seule ? | Oui   Non   Si non, combien de personne vous y compris, y-a-t-il dans votre foyer : __ |
| --- | --- |
| 39. Quelle est votre occupation actuelle ? | Exerce un emploi   Apprenti / Stage   Elève / Etudiant   Chômeur   Retraité  Préciser le dernier emploi : ___________  Au foyer   Congés parental temps plein   Autres  Préciser : ____________________ |
| 40. Quelle est votre catégorie socio professionnelle ? | Agriculteur / Exploitant   Artisan / Commerçant / chef d’entreprise   Cadre / Profession intellectuelle supérieure   Profession intermédiaire   Employé   Ouvrier   Sans activité professionnelle  |
| 41. Quel est votre niveau d’études ? | Jamais scolarisé   Maternelle / Primaire   Secondaire (collège/lycée)   Supérieur  |
| 42. Quelle est votre nationalité ? | Française   Autre  Précisez : ________________________ |
| 43. Quelle est la nationalité de vos parents ? | Française pour les deux parents   Autre nationalité pour au moins un des deux  |
| 44. Quel est votre code postal ? |  |
| 45. Actuellement avez-vous une couverture maladie ? | Sécurité sociale standard   CMU   AME   Absence de couverture maladie   Je ne sais pas  |
| 46. Bénéficiez-vous d’une couverture maladie complémentaire ? | Oui par CMU   Oui par mutuelle ou assurance privée   Oui mais je ne sais pas par quoi   Non aucune   Je ne sais pas  |

**Merci d’avoir répondu**. Nous tiendrons votre médecin informé au fur et à mesure de l’état d’avancement de l’étude et dès que possible des premiers résultats. N’hésitez pas à le contacter ou à nous contacter si vous désirez avoir des informations supplémentaires

**Etude Druti questionnaire «médecin»**

Nom et prénom du médecin : Département d’exercice :

Cachet du médecin Etiquette de numéro d’anonymat de la patiente

**1. Les informations concernant les symptômes de la patiente sont-elles reportées dans le registre ?**  Oui  Non 

Si non, merci de les compléter car ces informations sont primordiales pour l’étude.

**Description du cas**

2. Date d’apparition des symptômes : _ _ / _ _ / _ _ _ _ (jour / mois / année)

3. Terrain à risque d’infection urinaire compliquée :

| Anomalies organiques ou fonctionnelles de l’arbre urinaire | Non  | Oui  |
| --- | --- | --- |
| Grossesse en cours | Non  | Oui  |
| Pathologies chroniques  (par exemple : diabète, immunodépression, insuffisance rénale) | Non  | Oui  |
| Si oui la (ou les) quelles ?______________________________________________  __________________________________________________________________ | | |

4. Avez-vous réalisé une bandelette urinaire : Non Oui

5. Si oui, quel en a été le résultat (**- ou nombre de +**) ?

Leucocyturie : Nitrite : Hématurie :

Probabilité diagnostique :

| 6. Faites-vous le pari que l’ECBU de votre patiente sera positif ? | Oui  Non  |
| --- | --- |
| 7. La patiente, a-t-elle déjà eu une infection urinaire par le passé ? | Oui  Non  |
| 8. La patiente, pense-t-elle qu’il s’agit d’un épisode d’infection urinaire ? | Oui  Non  |

**Prise en charge de l’épisode actuel**

9. La patiente a-t-elle pris un traitement pour cet épisode de cystite avant la consultation : Non  Oui 

10. Si oui le ou lesquels ?_____________________________________________________________

11. Des examens complémentaires ont-ils été prescrits : Non  Oui 

12. Si oui, merci de cochez les cases correspondantes à ces examens :

ECBU (en sus de celui de l’étude) 

Echographie , Précisez la localisation : __________________________________

Autres examens  Le ou lesquels : ________________________________________

13. Traitement prescrit lors de cette consultation :

| Nom du médicament | Posologie | Durée |
| --- | --- | --- |
|  |  |  |
|  |  |  |
|  |  |  |
|  |  |  |

14. Avez-vous remis un arrêt de travail à la patiente ?

Non  Oui  14 bis. Si oui durée : _ _ jours

15. Hospitalisation au décours de la consultation :

Non Oui 

**Caractéristiques de la patiente**

16. A votre connaissance, la patiente a-t-elle eu une infection urinaire, au cours des 12 derniers mois ? (Ne prendre en compte que les épisodes datant de plus de 8 semaines)

Non  Oui 

17. **Si oui**, nombre d’infections urinaires au cours des 12 derniers mois : _ _

18. A votre connaissance, la patiente a-t-elle reçu une antibiothérapie (quelle qu’en soit l’indication), au cours des 3 derniers mois ?

Non  Oui  **Si oui, merci de compléter le tableau 1.**

19. A votre connaissance, un autre membre du foyer a-t-il reçu une antibiothérapie (quelle qu’en soit l’indication), au cours des 3 derniers mois ?

Non  Oui 

20. **Si oui**, nombre d’antibiothérapies pour l’ensemble des membres du foyer, au cours des 3 derniers mois : _ _

21. Au cours des 12 derniers mois, la patiente a-t-elle été hospitalisée (quelle qu’en soit l’indication, et longue ou courte durée) ?

Non  Oui   **Si oui, merci de compléter le tableau 2.**

22. **Si oui**, nombre d’hospitalisations au cours des 12 derniers mois : _ _

23. Au cours des 3 derniers mois, la patiente a-t-elle eu une consultation médicale ?

Non  Oui 

24. **Si oui**, nombre de consultation médicale au cours des 3 derniers mois : _ _

25. Au cours **du dernier mois**, la patiente a-t-elle eu un contact avec une personne hospitalisée ou vivant en institution (c-à-d hébergée dans une collectivité de type EHPAD, foyer pour personnes handicapées, …) ?

Non  Oui 

26. **Si oui**, merci de préciser les informations suivantes : (une ou plusieurs réponses possibles)

Contact privé occasionnel 

Contact professionnel répété (par exemple professionnel de santé) 

27. A votre connaissance et au cours **du dernier mois**, un des membres du foyer de la patiente a-t-il eu un contact avec une personne hospitalisée ou vivant en institution (c-à-d hébergée dans une collectivité de type EHPAD, foyer pour personnes handicapées, …) ?

Non  Oui 

28. **Si oui**, merci de préciser les informations suivantes : (une ou plusieurs réponses possibles)

Contact privé occasionnel 

Contact professionnel répété (par exemple professionnel de santé) 

29. Au cours du **dernier mois**, la patiente a-t-elle eu un sondage vésical ?

Non  Oui 

30. **Si oui**, préciser s’il s’agit :

d’un sondage à demeure 

d’un sondage intermittent 

31. Au cours des **12 derniers mois** la patiente a-t-elle voyagé à l’étranger ?

Non  Oui 

32. **Si oui**, nombre de voyages au cours des 12 derniers mois : _ _

**Et, merci de compléter le tableau 3.**

33. La patiente a-t-elle connaissance d’un diagnostic de portage de bactéries résistantes aux antibiotiques la concernant ?

Non  Oui  Ne sais pas 

34. Au cours des **12 derniers mois**, la patiente a-t-elle hébergée au moins une personne résidant habituellement à l’étranger ?

Non  Oui 

35. **Si oui**, dans quel(s) pays : ________________________

36. La patiente a-t-elle des contacts quotidiens avec des animaux ?

Non  Oui  **Si oui, merci de compléter le tableau 4.**

37. La patiente a-t-elle consommé de la viande crue au cours des trois derniers mois ?

Non  Oui  Ne sait pas 

**Tableau 1 : Informations relatives aux antibiothérapies**

Si vous avez répondu oui à la question 18, merci de compléter ce tableau

| Antibiothérapie n° | 1 | 2 | 3 | 4 | 5 | 6 |
| --- | --- | --- | --- | --- | --- | --- |
| Date de début | _ _ /_ _/_ _ | _ _ /_ _/_ _ | _ _ /_ _/_ _ | _ _ /_ _/_ _ | _ _ /_ _/_ _ | _ _ /_ _/_ _ |
| Antibiotique |  |  |  |  |  |  |
| Durée (cochez la case correspondant) |  ≤ 3 jours   4 à 7 jours   ≥ 8jours |  ≤ 3 jours   4 à 7 jours   ≥ 8jours |  ≤ 3 jours   4 à 7 jours   ≥ 8jours |  ≤ 3 jours   4 à 7 jours   ≥ 8jours |  ≤ 3 jours   4 à 7 jours   ≥ 8jours |  ≤ 3 jours   4 à 7 jours   ≥ 8jours |

**Tableau 2 : Informations relatives aux hospitalisations**

Si vous avez répondu oui à la question 21, merci de compléter ce tableau

| Hospitalisation n° | 1 | 2 | 3 | 4 | 5 | 6 |
| --- | --- | --- | --- | --- | --- | --- |
| Date de début | _ _ /_ _/_ _ | _ _ /_ _/_ _ | _ _ /_ _/_ _ | _ _ /_ _/_ _ | _ _ /_ _/_ _ | _ _ /_ _/_ _ |
| Durée |  |  |  |  |  |  |
| Passage en réanimation ou soins intensifs | Oui   Non   NSP  | Oui   Non   NSP  | Oui   Non   NSP  | Oui   Non   NSP  | Oui   Non   NSP  | Oui   Non   NSP  |

**Tableau 3 : Informations relatives aux voyages.**

Si vous avez répondu oui à la question 31, merci de compléter ce tableau

| Voyage n° | 1 | 2 | 3 | 4 | 5 | 6 |
| --- | --- | --- | --- | --- | --- | --- |
| Pays de destination |  |  |  |  |  |  |
| Durée |  |  |  |  |  |  |
| Date de départ (mois/année) | _ _ /_ _ _ _ | _ _ /_ _ _ _ | _ _ /_ _ _ _ | _ _ /_ _ _ _ | _ _ /_ _ _ _ | _ _ /_ _ _ _ |
| Contact avec le système de santé du pays | Oui   Non  | Oui   Non  | Oui   Non  | Oui   Non  | Oui   Non  | Oui   Non  |
| Si oui préciser : |  |  |  |  |  |  |
| Hospitalisation programmée | Oui   Non  | Oui   Non  | Oui   Non  | Oui   Non  | Oui   Non  | Oui   Non  |
| Hospitalisation en urgence | Oui   Non  | Oui   Non  | Oui   Non  | Oui   Non  | Oui   Non  | Oui   Non  |
| Dialyse | Oui   Non  | Oui   Non  | Oui   Non  | Oui   Non  | Oui   Non  | Oui   Non  |
| Passage en réanimation ou soins intensifs | Oui   Non  | Oui   Non  | Oui   Non  | Oui   Non  | Oui   Non  | Oui   Non  |

**Tableau 4 : Informations relatives aux animaux.**

Si vous avez répondu oui à la question 36, merci de compléter ce tableau, **préciser pour chaque type d’animaux**

| Type d’animaux |  |  |  |  |  |
| --- | --- | --- | --- | --- | --- |
| Nombre |  |  |  |  |  |
| Type de contact | Professionnel   Compagnie  | Professionnel   Compagnie  | Professionnel   Compagnie  | Professionnel   Compagnie  | Professionnel   Compagnie  |
| Un de ces animaux a-t-il reçu des antibiotiques au cours **des trois derniers mois** | Oui   Non   Ne sait pas  | Oui   Non   Ne sait pas  | Oui   Non   Ne sait pas  | Oui   Non   Ne sait pas  | Oui   Non   Ne sait pas  |

**Attention partie sur les caractéristiques socio-économiques en page 4. Merci de tourner la feuille.**
