# Supplementary material for: Factors associated with the duration of symptoms in adult women with suspected cystitis in primary care
Source: PLoS One. 2018 Jul 25;13(7):e0201057. doi: 10.1371/journal.pone.0201057 (PMC6059455; doi:10.1371/journal.pone.0201057)
Supplement: S3 Table — (DOC) [file pone.0201057.s003.doc]

**Etude Druti questionnaire de suivi** (à remettre à la patiente, si elle accepte le suivi)

Nom et prénom du médecin : Département d’exercice :

Cachet du médecin Etiquette de numéro d’anonymat de la patiente

Afin de préparer ces entretiens téléphonique, pourriez-vous conserver les informations concernant vos dépenses de santé (boites de médicaments, ordonnance, compte rendu d’examen ou d’hospitalisation)

**Evolution des symptômes les deux premières semaines**

1. Merci d’indiquer au fur et à mesure l’évolution de vos symptômes, durant les deux semaines après la consultation.

| Jour après votre 1^ère^ consultation | 1 | 2 | 3 | 4 | 5 | 6 | 7 | 8 | 9 | 10 | 11 | 12 | 13 | 14 |
| --- | --- | --- | --- | --- | --- | --- | --- | --- | --- | --- | --- | --- | --- | --- |
| Êtes-vous toujours gênée ? | Oui   Non  | Oui   Non  | Oui   Non  | Oui   Non  | Oui   Non  | Oui   Non  | Oui   Non  | Oui   Non  | Oui   Non  | Oui   Non  | Oui   Non  | Oui   Non  | Oui   Non  | Oui   Non  |

**Entretien à 2 semaines**

2. Votre médecin avait-t-il prescrit des examens complémentaires lors de votre première consultation?

Non  Oui 

3. Si oui, quels examens avez-vous effectué ? .................................

4. Avez-vous acheté les médicaments prescrits par votre médecin lors de votre première consultation?

Non  Oui 

5. Si oui, quels médicaments avez-vous acheté ? .................................

6. **Si** votre médecin vous a prescrit un antibiotique, avez-vous pris la totalité de votre traitement ?

Non  Oui 

7. Pour ce problème urinaire, avez-vous pris d’autres traitements que ceux prescrits par votre médecin, y compris des médicaments achetés sans ordonnance?

Non  Oui 

8. Si oui, lesquels ? .................................

9. Pour ce problème urinaire avez-vous eu besoin de consulter un médecin une nouvelle fois ?

Non  Oui 

10. Si oui, merci de compléter le tableau suivant :

| Type de médecin (généraliste/ autre spécialité) | Nombre de consultations  (0 si aucune) |
| --- | --- |
| Le médecin généraliste vu lors de la première consultation | _ _ |
| Un autre médecin généraliste | _ _ |
| Un médecin d’une autre spécialité.  Précisez quelle spécialité : ........................... | _ _ |

10 bis. Le médecin vous a-t-il prescrit des médicaments ? Non  Oui 

Si oui, quels médicaments avez-vous acheté ?

11. Pour cette infection urinaire depuis la consultation initiale, a-t-il été nécessaire de réaliser des examens non prescrits lors de la consultation initiale ?

Non  Oui 

12. Si oui lesquels ? .................................

13. Avez-vous été hospitalisée depuis la première consultation ?

Non  Oui 

14. Si oui, merci de préciser les dates, le service et les raisons de cette hospitalisation : ..................................................................

..................................................................

15. Pour ce problème urinaire, avez-vous manqué un ou plusieurs jours de travail ?

Non  Oui 

16. Si oui, 15. Combien de jours : __ __

17. Et avez-vous bénéficié d’un arrêt de travail par votre médecin ? Non  Oui 

18. Si oui combien de jours : __ __

**Entretien à 8 semaines**

19. Au cours des 6 dernières semaines, avez-vous eu de nouveaux signes urinaires ?

Non  Oui 

**Si vous avez répondu non à la question 19, merci de répondre directement à la question 31.**

**Si vous avez répondu oui à la question 19, merci de répondre aux questions suivantes.**

20. Avez-vous eu besoin de consulter un médecin pour ces nouveaux signes ?

Non  Oui 

21. Si oui, merci de compléter le tableau suivant :

| Type de médecin (généraliste/ autre spécialité) | Nombre de consultations  (0 si aucune) |
| --- | --- |
| Le médecin généraliste vu lors de la première consultation | _ _ |
| Un autre médecin généraliste | _ _ |
| Un médecin d’une autre spécialité.  Précisez quelle spécialité : ........................... | _ _ |

22. Avez-vous pris un traitement pour votre problème urinaire, y compris des médicaments achetés sans ordonnance?

Non  Oui 

23. Si oui lesquels? ................................

24. A-t-il été nécessaire de réaliser de nouveaux examens pour ces nouveaux signes urinaires ?

Non  Oui 

25. Si oui lesquels? ................................

26. Avez-vous été hospitalisée à cause de ce nouveau problème urinaire ?

Non  Oui 

27. Si oui, merci de préciser les dates, le lieu et les raisons de cette hospitalisation : ..................................................................

..................................................................

28. Avez-vous manqué un ou plusieurs jours de travail à cause de ces nouveaux signes urinaires ?

Non  Oui 

29. Si oui, 28. Combien de jours : __ __

29. Et avez-vous bénéficié d’un arrêt de travail par votre médecin ?

Non  Oui

30. Si oui, combien de jours : __ __

**31. Seriez-vous d’accord pour être recontactée dans 12 mois afin de poursuivre notre recherche sur les infections urinaires ?**

Non  Oui 
